# Schisandra Inhibit Bleomycin-Induced Idiopathic Pulmonary Fibrosis in Rats via Suppressing M2 Macrophage Polarization

**DOI:** 10.1155/2020/5137349

**Published:** 2020-08-20

**Authors:** Zhaojuan Guo, Siru Li, Nan Zhang, Qianjun Kang, Huaqiang Zhai

**Affiliations:** ^1^Beijing Institute of Traditional Chinese Medicine, Beijing University of Chinese Medicine, Beijing 100029, China; ^2^Liberal and Art College, New Jersey Institute of Technology, Newark 07102, USA; ^3^School of Chinese Materia Medica, Beijing University of Chinese Medicine, Beijing 102488, China

## Abstract

Idiopathic pulmonary fibrosis (IPF) is defined as a specific form of chronic, progressive fibrosing interstitial pneumonia of unknown cause and limited to the lungs. *Schisandrae chinensis fructus* (Wuweizi, Schisandra) is commonly used traditional Chinese medicines (TCM) for the treatment of pulmonary fibrosis, bronchitis, and other lung diseases in China. In this study, we investigated the therapeutic effect of Schisandra on IPF which is induced by bleomycin (BLM) in rats and the inhibition of alternatively activated macrophage (M2) polarization. Bleomycin-induced pulmonary fibrosis was used as a model for IPF, and rats were given drug interventions for 7 and 28 days to evaluate the role of Schisandra in the early oxidative phase and late fibrotic phases of BLM-induced pulmonary injury. The data showed that Schisandra exerted protective effects on BLM-induced pulmonary injury in two phases, which were improving inflammatory cell infiltration and severe damages of lung architectures and decreasing markers of M2 subtype. In order to prove the inhibitory effect of Schisandra on M2 polarization, in vitro experiments, we found that Schisandra downregulated the M2 ratio, which confirmed that the polarization of M2 was suppressed. Moreover, Schisandra blocked TGF-*β*1 signaling in AMs by reducing the levels of Smad3 and Smad4; meanwhile, the upregulation of Smad7 by Schisandra also promoted the effect of inhibition on the TGF-*β*1/Smad pathway. These results demonstrate that suppression of M2 polarization by Schisandra is associated with the development of IPF in rats.

## 1. Introduction

IPF is a chronic progressive disease with poor prognosis characterized by interstitial pneumonia. During the process of IPF, the secretion of a large amount of proinflammatory cytokines and the deposition of collagen in the extracellular matrix, as well as the abnormal repairment of alveolar epithelial cells, lead to the scarring of the lungs and irreversible loss of function [1–3]. As the pathogenesis of IPF remains unclear, its high morbidity and mortality make the treatment of IPF even more knotty [4].

Alveolar macrophages (AMs) are the main effector cells of the immune response during the process of IPF, which reside in the alveoli and have the dual properties of proinflammation and anti-inflammation. The spatiotemporal dynamics of the number and function of pulmonary macrophages are closely related to the course and condition of pulmonary fibrosis whether the lung disease is undergoing acute progression due to known etiology or idiopathic occult progression [5, 6]. Macrophage polarization changes dynamically and manifests different functional phenotypes in different microenvironments. Macrophages are classified into M1 and M2 types according to their phenotypes and secreted cytokines [7, 8]. Phenotypically, M2 macrophages are characterized by low levels of CD86, MHCII, and iNOS with high levels of arginase-1 and macrophage mannose receptor (CD206) [9, 10]. The polarization subtype of macrophages corresponds to the early alveolitis stage and advanced fibrosis stage of IPF [11, 12]. Previous studies have revealed that molecular entities participate in the selectively activated macrophage and cytokines produced by M2 macrophages promote fibrosis, which contributes to the persistence of fibrotic microenvironment [13–15]. This phenomenon suggests that M2 is a potential therapeutic target for IPF. As a potent and long-acting glucocorticoid, dexamethasone (Dex) can suppress the expressions of inflammatory cytokines, such as TNF-*α*, TGF-*β*1, and platelet-derived growth factor (PDGF) in AMs, and downregulate the mRNA of monocyte chemotactic protein-1 (MCP-1) [16–19].

In recent years, TCM and their formulations have been shown to exhibit great efficacy in treating lung fibrosis with good prospects for application [20–23]. Schisandra, the fruit of *Schisandra chinensis (Turcz.) Baill.*, is a traditional Chinese herbal medicine, which is widely used to treat cough and dyspnea in China [24]. Modern pharmacological studies show that Schisandra exhibits therapeutic effects of anti-inflammation, hepatoprotection, and antioxidant properties [25, 26]. Some researchers exposed that plenty of bioactive compounds of Schisandra, such as deoxyschizandrin, schisandrin B, and schizandrol, play a role of immune regulation in lung and kidney diseases [27, 28]. Our previous study found that the aqueous extract of Schisandra has preventive and therapeutic effects on pulmonary fibrosis in rats with IPF, suppressing the expressions of monocyte chemotactic protein 1 (MCP-1) and macrophage inflammatory protein-l*α* (MIP-1*α*). Schisandra inhibited the angiogenesis in the lung and downregulated the vascular endothelial growth factor (VEGF), TGF-*β*1, endothelin-1 (ET-1), and the proangiogenic factors of Angiopoietin-2 (Ang-2) [29–32]. The polarization of M2 changes dynamically at different stages of IPF, while the effect of Schisandra on M2 polarization and the related signaling mechanisms are unclear. Therefore, this study is aimed at investigating Schisandra's protective effect on IPF and inhibitory effect on M2 polarization, providing a useful reference for expanding the clinical application of Schisandra.

## 2. Materials and Methods

### 2.1. Animals and Drugs

A total number of 89 Wistar male rats (age, 6-8 weeks; weight, 200 ± 20 g) were obtained from Beijing Vital River Laboratory Animal Technology Co., Ltd. (Permission No. SCXK (Beijing) 2012-0001). All animals were housed individually with a 12 h/12 h light/dark cycle and had free access to food and water. The experimental procedures were approved by the Ethical Committee on Animal Research at Beijing University of Chinese Medicine and conducted following the Guide for the Care and Use of Laboratory Animals established by the US National Institutes of Health.

Schisandra (Batch No. 1502087) was purchased from Tongrentang Chinese Pharmaceutical Co. Ltd. (Beijing, China), which was identified by Prof. Huaqiang Zhai. The quality of Schisandra is in accord with the standard of Pharmacopoeia of People's Republic of China 2015 Edition. Boiling Schisandra seeds in 8 and 4 times water of the weight of drug for 2 h each time to prepare the Schisandra's decoction concentrated to 1 gram decoction per milliliter, and the extractions were stored at 4°C. Dexamethasone acetate injection is at 5 ml/kg (0.15 mg/ml, Tianjin Lisheng Pharmaceutical Co. Ltd., Tianjin, China; Batch No. 1410022). Moreover, bleomycin HCL (Nippon Kayaku Co. Ltd., Japan; Batch No. 440392; Import Drug Registration Certification No. H20090885) was used to induce pulmonary fibrosis.

### 2.2. Drug Administration

In vivo experiments, after a week of adjustable feeding, 64 rats were divided into the sham, model, Schisandra, and dexamethasone (hereinafter referred to as Dex) groups with 16 rats of each randomly. After anesthetized with 30 mg/kg pentobarbital sodium, the rats were intratracheally injected with 5 mg/kg bleomycin via tracheostomy to induce pulmonary fibrosis [33], and the rats in the sham group received an intratracheal injection of the same volume of normal saline. After surgery, rats from the model group and the sham group were injected with normal saline, and the rats in the model group received daily intragastric administration consecutively with Schisandra decoction (5 mg/kg/d) or Dex (0.5 mg/kg/d). All animals were guaranteed to be given an equal volume of drug or solvent. Eight rats in each group were sacrificed on the 7^th^ and 28^th^ day. The lung tissues were rapidly excised after the sacrifice of rats, and the left lung was reserved for hematoxylin-eosin staining (HE) and immunohistochemistry staining.

### 2.3. Drug-Containing Serum Preparation

We divided rats into the sham group and the Schisandra group randomly, and there were five rats in each group. Rats in the Schisandra group rats received daily intragastric administration consecutively for seven days with Schisandra decoction (5 mg/kg/d) to obtain the drug-containing serum. Water but not feed was provided on the sixth day for 12 h. Then, all the rats were euthanized with sodium pentobarbital; blood in the abdominal aorta was sucked out. The blood samples were centrifuged at 3500 rpm for 10 min. Subsequently, the upper serum was separated and inactivated by incubation in a water bath at 56°C for 30 min. Then, serum was filter sterilized using a microporous membrane with 0.22 *μ*m pores and finally stored in aliquots at -80°C.

### 2.4. Histopathological Examination

The left lung samples were fixed in 10% formalin buffer and cut into 3 mm pieces, and then, it was used for HE and immunohistochemistry staining. The pathological morphology changes in lung tissue were observed with H&E staining under an ordinary light microscope (Nikon microscope, ECLIPSELV100POL/50IPOL, Japan). The grades of lung fibrosis in Szapiel et al.'s [34] were used to evaluate the pathological morphology and its classification. “(0)” indicates no obvious alveolar rupture and fusion on the stage of alveolitis (Day 7); “(+)” indicates mild alveolar rupture and fusion; “(++)” indicates moderate alveolar rupture and fusion; “(+++)” indicates severe alveolar rupture and fusion; on the stage of alveolitis (Day 28), “(0)” indicates no obvious fibrous tissue hyperplasia; “(+)” indicates mild fibrous tissue hyperplasia; “(++)” indicates moderate fibrous tissue hyperplasia; “(+++)” indicates severe fibrous tissue hyperplasia.

### 2.5. Immunohistochemistry Analysis for CD163

Immunohistochemistry analysis was performed by using the streptavidin-biotin-immunoperoxidase technique. 8% normal goat serum was used to block the sections for 2 h, after which they were treated with primary antibodies specific for CD163 (1 : 500, anti-rabbit CD163, Abcam, USA) in a humidified chamber overnight at 4°C. Sections were washed with PBS 3 times and incubated with goat anti-rat secondary antibodies at room temperature for 15 min. We examined all sections blindly at ×40 magnification by using a light microscope (Olympus CX21, Japan). The stained cells or stained area percentage was counted or quantified with Image-Pro Plus 6.0 (Media Cybernetics, Inc., Rockville, MD, USA); each section was photographed at a 400x, ensuring that the background light of each photo was consistent. Image-Pro Plus 6.0 was used to select the same brown color as the unified standard for judging all photos. Each photo was analyzed to obtain the average optical density (MOD) and cumulative optical density (IOD SUM) of each slice.

### 2.6. Cell Culture

Alveolar macrophages were obtained from bronchoalveolar lavage fluid (BALF) in 10 rats, which was performed by lavaging the lung eight times with 10 ml of saline each via a tracheal catheter [32, 35]. BALF was centrifuged with 1,500 rpm at 4°C for 15 min. The supernatants were discarded, and the precipitate was washed twice with PBS and centrifuged. The supernatants were discarded again. Then, the erythrocyte lysate was added, and the mixture was centrifuged at 500 rpm for 5 min. The supernatants were discarded, and the cell pellet was washed once with PBS and centrifuged. The supernatants were removed; then, all the cells were suspended in RPMI-1640 supplemented with 10% (*v*/*v*) fetal bovine serum (FBS, Invitrogen, USA) 1% (*v*/*v*) penicillin and streptomycin (hereafter referred to as RPMI-1640 medium) [36]. The cell suspension was seeded into each well of a 12-well plate at a density of 5 × 10^5^ cells per well, and cells were cultured in a humidified incubator with 5% CO_2_ at 37°C for 2 h. Then, the supernatants were removed. 1 ml RPMI-1640 medium was added to each well. Finally, the cell density of AMs was adjusted to 1 × 10^6^/ml/well. Then, 100 *μ*l of the cell suspension was added into each well of a 96-well plate, which was divided into 12 groups averagely [37]. After incubation for 2 h, each well was added 100 *μ*l of RPMI-1640 medium with different concentrations of serum containing Schisandra (10%, 20%, 30%, and 40%); blank serum was added to blank group cells. Finally, all cells were cultured in 5% CO_2_ at 37°C for 48 h.

### 2.7. MTT Assay

Conventional MTT assay was used to evaluate the cytotoxic effect of Schisandra. AMs (1 × 10^5^ cells/well) were seeded and plated into 96-well plates, then incubated in serum-free medium for 2 h. After appropriate treatment according to the experimental grouping, the cells were incubated with Schisandra medicated serum (10%, 20%, 30%, and 40%, *v*/*v*) for 48 h. Subsequently, each well was added to 20 *μ*l of MTT (5 mg/ml, Solarbio, China), and the cells were incubated for an additional 4 h. Then, the supernatants were removed, and the formazan crystals were dissolved in DMSO (100 *μ*l, Sigma, USA). Finally, absorbance was measured at 570 nm using a SPECTROstar Omega Microplate Reader (BMG Labtech, Germany).

### 2.8. Cell Polarization

After primary AMs were cultured in RPMI-1640 medium for 4 h, cell concentration was adjusted to 3 × l0^6^/ml/well; then, cells were stimulated with IL-4 (10 ng/ml, PrimeGene, China) for 48 h to promote M2 polarization. Other primary AMs (3 × l0^6^/ml, 6-well plates) were cultured in RPMI-1640 medium with 10% (*v*/*v*) of Schisandra serum, respectively, for 48 h to promote macrophage polarization.

### 2.9. Flow Cytometry

Macrophages were collected and labeled with the markers of M2 (FITC-conjugated CD206, Abcam, USA) and markers of AMs (APC-conjugated CD11b, BD, USA), and PE-conjugated IgG was used as isotype controls. Cells (3 × l0^6^ cells/well, 6-well plates) were mixed with isotype-matched IgG as negative controls or monoclonal antibodies at 4°C for 30 min in the dark. After incubation, cells were washed with cold PBS 3 times and cell phenotype was detected by FACS analysis.

### 2.10. Enzyme-Linked Immunosorbent Assay (ELISA)

Active TGF-*β*1 expression in the cell culture media was measured using ELISA kits according to the manufacturer's protocol (MultiSciences Lianke Biotech Co., Ltd.). The optical density (OD) was measured at 450 nm by a microplate reader (MULTISKAN MK3, Thermo, USA).

### 2.11. Western Blot Analysis

The Western blot method was used to determine the expressions of Smad3, Smad4, and Smad7. Cell suspension of AMs was homogenized in ice-cold radioimmunoprecipitation (RIPA) lysis buffer (Beyotime Institute of Biotechnology, Haimen, China). Cell debris was cleared by centrifugation for 10 mins at 12,000 rpm under 4°C, and protein concentrations were determined using a Bicinchoninic Acid (BCA) Protein Assay Kit (Cwbiotech, China). Bovine serum albumin was used as the standard. Protein concentrations were transferred electrophoretically onto polyvinylidene difluoride (PVDF) membranes, and then, the blotted membranes were blocked with 5% nonfat dry milk (*w*/*v*) in Tris-buffered saline with 0.1% Tween-20 (TBS-T) for 1 h at room temperature. After that, the blots were incubated overnight at 4°C with primary antibodies: Smad3 (1 : 5000, Abcam, USA), Smad4 (1 : 1000, Abcam, USA), and Smad7 (1 : 500, Abcam, USA). After washing with TBST, the membranes were incubated with horseradish peroxidase-labeled goat anti-mouse IgG (1 : 10000, Jackson ImmunoResearch, USA) or goat anti-rabbit IgG (10000, Jackson ImmunoResearch, USA) for 1 h and washed 3 times with TBST again. Densitometry analysis was analyzed using the Gel Image System ver.4.0 (Tanon, China).

### 2.12. Real-Time Quantitative Polymerase Chain Reaction (RT-qPCR)

Total RNA was isolated from macrophages using a Trizol reagent (Invitrogen, Carlsbad, USA). Total RNA was reverse-transcribed (RT) to cDNA using a reverse transcriptional polymerase chain reaction (RT-PCR) kit (KAPA Biosystems, USA) per the manufacturer's instructions. Subsequently, PCR was performed on the resulting cDNA with an SYBR PCR Mixture on an RT-PCR detection system. The primers used for RT-PCR were as follows: Smad4: 5′-CCAGTACCACCAACTTCCCC-3′ and 5′-TCCATTCTGCTGCTGTCCTG-3′; Smad3: 5′-AATGTCTCCCCAACTCGCTG-3′ and 5′-CGACCACCAGATGAACCACA-3′; Smad7: 5′-CGGAAGTCAAGAGGCTGTGT-3′ and 5′-CGTCTGGACAGTCTGCAGTT-3′; *β*-actin: 5′-CTTCCAGCCTTCCTTCCTGG-3′ and 5′-AATGCCTGGGTACATGGTGG-3′. The experiments of MTT assay, flow cytometry, ELISA, Western blot analysis, and RT-qPCR were repeated three times in parallel.

### 2.13. Statistical Analysis

The data were presented as the mean ± SD. Statistical analysis was performed using SPSS 25.0 software (SPSS, Chicago, USA). The difference between the two groups was compared with a *t*-test. Statistical comparison of multiple groups was performed by one-way ANOVA. Results were considered significant at the two-sided *P* level of 0.05.

## 3. Result

### 3.1. Severity Classification of Alveolitis and Pulmonary Fibrosis

**Figure 1:**
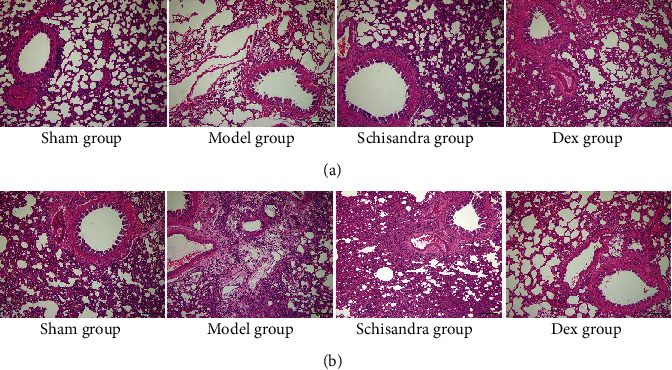
Schisandra suppressed BLM-induced pulmonary fibrosis in rats. After the establishment and pathological evolution of a rat model of pulmonary fibrosis by tracheal BLM (5 mg/kg) instillation, the rats in the sham group received an intratracheal injection of the same volume of normal saline. Lung sections were stained with HE for histological assessment, and representative images are shown. (a) HE staining of the pulmonary tissue on Day 7 (*n* = 8). (b) HE staining of the pulmonary tissue on Day 28 (*n* = 8). *n* = 8 per group. Scale bar: 200 *μ*m.

HE staining results showed clear morphologic changes, including alveolar destruction and inflammatory cell infiltration of the interstitium in lung tissues of the model group on Day 7 (Figure 1(a)). However, the Schisandra-treated group improved inflammatory cell infiltration and severe damages of lung architectures, which could be seen from Table 1. On Day 28, visible fibrous tissue hyperplasia was observed in the model group, the fusion of alveoli, apparent increases in interalveolar septal thickness, and the collapse of many alveoli cells, while these pathological alterations in the lung tissues reduced following Schisandra treatment (Figure 1(b)). Our previous studies have shown that the main pathological manifestations of IPF were alveolitis on Day 7; fibrous tissue proliferation was less. Fibrous bundles showed significant proliferation on Day 28. This result is also consistent with our previous research. The severity classification of alveolitis and pulmonary fibrosis is shown in Table 1.

### 3.2. Levels of the Immunohistochemical Staining for CD163 in Lung Tissues

**Figure 2:**
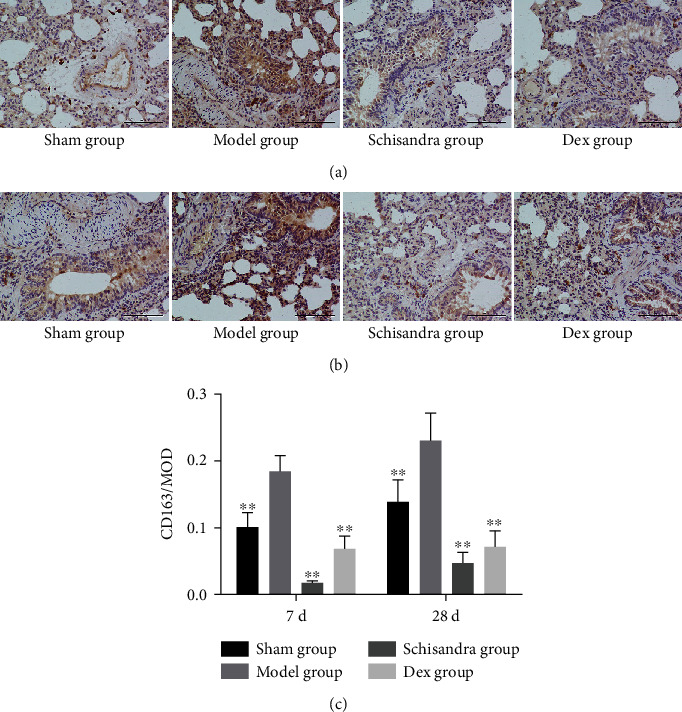
Schisandra inhibited CD163 expression in vivo. (a) Immunohistochemical staining for CD163 in lung tissues of different experimental groups on Day 7. (b) Immunohistochemical staining for CD163 in lung tissues of different experimental groups on Day 28. (c) Optical density for CD163-positive tissues in different experimental groups. *n* = 8 per group. Scale bar: 200 *μ*m. Data were represented as mean ± SD. ^∗^*P* < 0.05 and ^∗∗^*P* < 0.01 compared with the model group.

Several attempts prove that M2 macrophages could increase CD163 expression and have anti-inflammatory and tissue repair properties [38]. Thus, we measured the expression of CD163 in BLM-induced pulmonary fibrosis. Our experimental results showed that lung tissue in the model group revealed prominent brown CD163 staining on Day 7; however, Schisandra inhibited these increase (Figure 2(a)). On Day 28, the CD163 in the model group showed a more production than that on Day 7 (Figure 2(b)); this suggested that CD163 expression continues to increase with the progress of pulmonary fibrosis, while the Schisandra group showed a lower CD163 level (Figure 2(c)). As the characteristic active markers, CD163 characterizes the activation state of the M2 phenotype [39]. It can be seen from our data that Schisandra inhibited the CD163 level during the progress of pulmonary fibrosis; we speculate that the inhibition of IPF by Schisandra is related to M2 polarization.

### 3.3. Effect of Schisandra on the Viability of AMs

**Figure 3:**
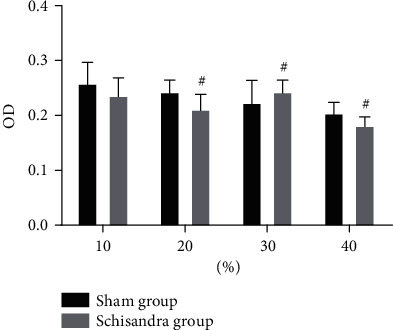
Effect of Schisandra on the viability of AMs. After cells were cultured with Ephedra and Schisandra for 48 h, cell viability was detected using MTT assays. Data were represented as mean ± SD, *n* = 6 per group. ^#^*P* < 0.05 and ^##^*P* < 0.01 compared with the blank group.

The effect of the serum containing Schisandra on AMs was assessed by the MTT method. MTT assays showed that 10% (*v*/*v*) Schisandra-medicated serum induces no observable cell cytotoxicity under the tested concentrations (Figure 3). Thus, this concentration was used in subsequent experiments.

### 3.4. Schisandra Downregulated the M2 Polarization

**Figure 4:**
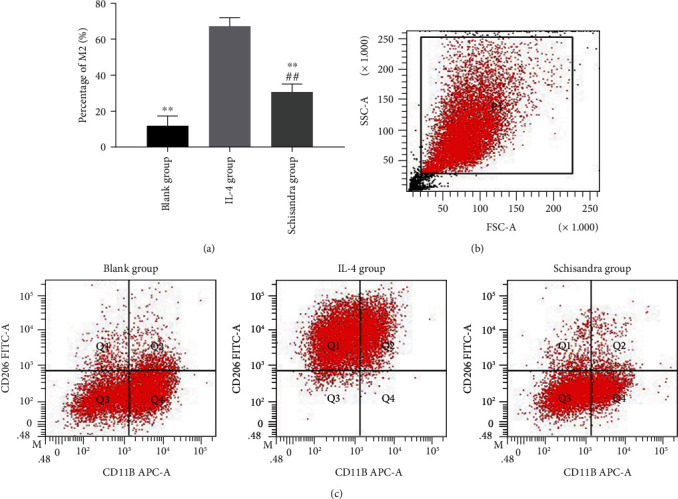
Schisandra inhibited the polarization of AM to M2 subtype in vitro. The anti-CD11b-APC antibody and anti-CD206-FITC antibody identified AMs and M2, and the percentage of M2 was then determined by flow cytometry. (a) Percentage of M2 subtype in all groups. (b) Representative gates of forwarding and side scatter. (c) Flow cytometry analysis of cell surface markers on alveolar macrophages. Data were represented as mean ± SD, *n* = 6 per group. ^#^*P* < 0.05 and ^##^*P* < 0.01 compared with the blank group, ^∗^*P* < 0.05 and ^∗∗^*P* < 0.01 compared with the IL-4 group.

Our other interesting finding was that Schisandra suppressed M2 polarization (Figures 4(a)–4(c)). The ratio of M2 in the Schisandra group was 30.73%, while it was 67.17% in the IL-4 group (Figure 4(b)). The number of M2 phenotype in the Schisandra group was significantly lower than that of the IL-4 group. These results indicated that AMs polarized to the M2 subtype were suppressed by Schisandra.

### 3.5. Schisandra Decreased the TGF-*β*1

**Figure 5:**
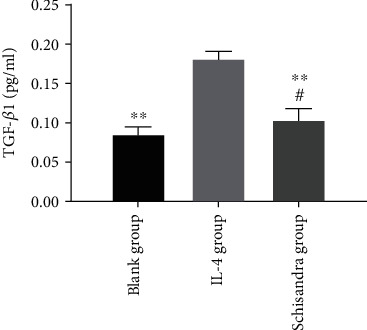
The level of TGF-*β*1 in cell culture supernatants was evaluated by ELISA. Data were represented as mean ± SD, *n* = 6 per group. ^#^*P* < 0.05 and ^##^*P* < 0.01 compared with the blank group, ^∗^*P* < 0.05 and ^∗∗^*P* < 0.01 compared with the IL-4 group.

A previous study found that the M2 phenotype increased the TGF-*β*1 level and contributed to the fibroproliferative repair in the late IPF [40]. To determine whether Schisandra exerts its antifibrotic effects by inhibiting TGF-*β*1, we measured TGF-*β*1 activity in the cell culture fluid. According to the results of ELISA, in contrast to normal cells, IL-4-treated cells upregulated the TGF-*β*1 level (Figure 5); however, TGF-*β*1 production was significantly suppressed when AMs were cultured with conditioned medium from AMs treated with Schisandra. These results suggested that Schisandra inhibited AMs polarized to M2 might be related to the TGF-*β*1 level.

### 3.6. Schisandra Inhibited the Activities of Smad3 and Smad4 and Upregulated the Smad7

**Figure 6:**
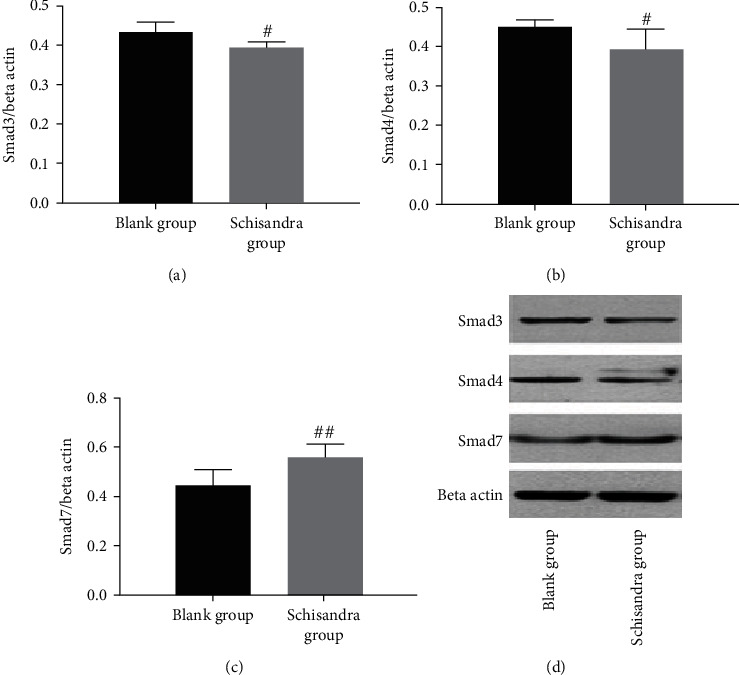
Expressions of Smad3, Smad4, and Smad7 in AMs were detected using Western blot analysis after cells were cultured with Ephedra and Schisandra for 48 h. (a) Smad3. (b) Smad4. (c) Smad7. (d) Smad3, Smad4, and Smad7 protein expressions in lung tissues in each group. Data were represented as mean ± SD, *n* = 3 per group. ^#^*P* < 0.05 and ^##^*P* < 0.01 compared with the blank group.

TGF-*β*1/Smad signaling has been demonstrated to contribute to tissue fibrosis process, including lung fibrosis, cardiac fibrosis, and kidney fibrosis [41–44]. Phosphorylations of Smad3 and Smad4 are the major regulators of the initiation of TGF-*β*1 signal transduction. Having shown that Schisandra could suppress cell polarization and that Schisandra could block TGF-*β* signaling, we next sought to investigate whether the TGF-*β*/Smad signaling pathway mediates the suppression of polarization by Schisandra in M2. The results of Western blot showed that Schisandra reduced the levels of Smad3 and Smad4 (Figures 6(a) and 6(b)). TGF-*β*1 has shown a function in promoting cellular differentiation from fibroblast to myofibroblast [45]. Once activated, the ligand, such as TGF-*β*1, binds to its receptors in the cellular membrane; phosphorylated Smad3 forms a complex with Smad4, activating transcription of target genes in the downstream of the TGF-*β*1/Smad signaling pathway. Therefore, elevated phosphorylated Smad3 level is regarded as the activation of the TGF-*β*1/Smad signaling pathway. In contrast, Smad7 is a negative regulator of the TGF-*β*1/Smad signaling pathway; Smad7 competes with Smad2 and Smad3 for binding to T*β*RI, which in turn interferes with the combination of Smad2 and Smad3 with Smad4, and inhibits the transmission of TGF-*β*1 signaling into cells [46, 47]. In this study, the Schisandra treatment group led to a significant increase in the expression of Smad7. This result suggests that Schisandra could block TGF-*β*1 signaling by reducing the levels of Smad3 and Smad4 and increasing Smad7.

### 3.7. Schisandra Suppressed the Expressions of Smad3 and Smad4 mRNA and Upregulated the Smad7 mRNA

**Figure 7:**
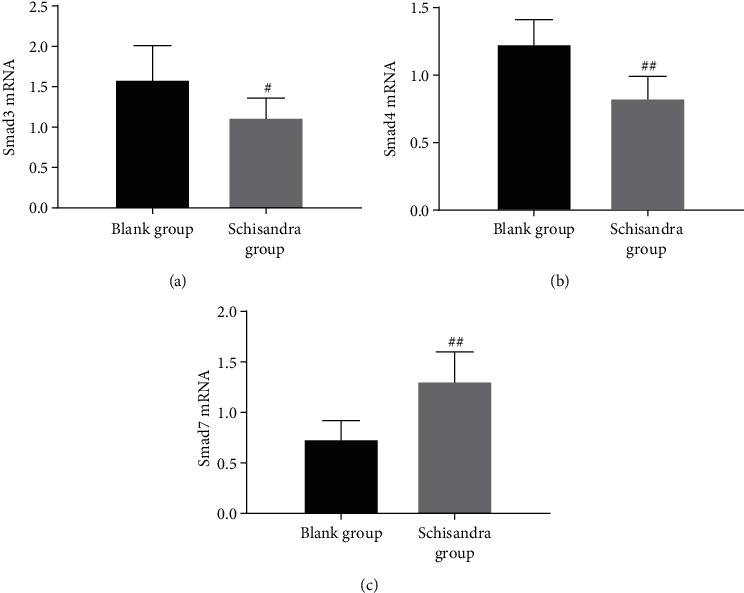
The protein levels of Smad3, Smad4, and Smad7 were determined using RT-PCR after cells were cultured with Ephedra and Schisandra for 48 h. (a) Smad3. (b) Smad4. (c) Smad7. Data were represented as mean ± SD, *n* = 3 per group. ^#^*P* < 0.05 and ^##^*P* < 0.01 compared with the blank group.

To confirm whether Schisandra exerts its antifibrotic effects by blocking the TGF-*β*1/Smad pathway, we evaluated Smad3, Smad4, and Smad7 mRNA levels in AMs by RT-PCR. Treatment with Schisandra markedly inhibited Smad3 and Smad4 mRNA compared with the blank group (Figures 7(a) and 7(b)); however, Smad7 mRNA was upregulated by Schisandra (Figure 7(c)), as shown by Western blot and RT-PCR analyses. Our results suggest that Schisandra inhibits polarization of M2 and this effect is related to the TGF-*β*/Smad signaling.

At present, there is no recognized cell model of IPF in vitro; therefore, we only observed the effects of test drugs on Smad3, Smad4, and Smad7 target proteins of normal AMs to prove the repression of Schisandra on them. With the further study of the pulmonary fibrosis model in vitro, we will carry out further investigations to prove the inhibitory effect of Schisandra on the TGF-*β*1/Smad pathway in rats with BLM-induced pulmonary fibrosis.

## 4. Discussion

The present study evaluated the effect of Schisandra on M2 phenotype in bleomycin-induced pulmonary fibrosis rats. Based on our previous research, this study deeply analyzed the polarization tendency of Schisandra on AM in IPF rats and interpreted its mechanism from the tissue, cell, and molecular levels. In vivo, it was shown that Schisandra improved the pathological morphology of lungs in IPF rats and reduced fusion of pulmonary alveoli, inflammatory cell infiltrates, which is consistent with the results of our previous studies. Besides, Schisandra downregulated the CD163 (M2 marker) level. Subsequently, we investigate whether Schisandra inhibited the development of IPF by inhibiting polarization of M2; in vitro experiments, we found that Schisandra downregulated the M2 ratio, which confirmed that the polarization of M2 was suppressed. Besides, the attenuation of TGF-*β*1 by Schisandra downregulated signal conduction of Smad3 and Smad4, while upregulation of Smad7 blocked the signal transduction of the TGF-*β*1/Smad pathway. It indicated that Schisandra inhibited the IPF process by suppressing M2 polarization, and that is related to the TGF-*β*1/Smad pathway.

Smads are the key receptors for the TGF-*β*1/Smad pathway. The Smads family is classified into three categories based on structure and function: the receptor-regulated Smads (R-Smads), the common Smads (Common Smads, C-Smads), and the inhibitory Smads (I-Smads) [48, 49]. R-Smads include Smad 1, Smad2, Smad3, Smad5, and Smad8. C-Smads include Smad4 alone, and Smad6 and Smad7 belong to I-Smads. As the signal transduction protein of TGF-*β*, R-Smads bind to membrane-bound serine/threonine receptors and be phosphorylated, then activated by its kinase activity. C-Smads bind to the activated R-Smads to form an oligomer and are transported to the nucleus. I-Smads include Smad7, which are negatively regulated in the TGF-*β*1/Smad pathway by competing with Smad3 to bind to TGF-*β* receptor type I (T*β*RI), inhibiting the activation in this pathway [50–54]. The evidence demonstrates that TGF-*β*1/Smad plays a critical role during pulmonary fibrogenesis [55–57]. TGF-*β*1, Smad2, and Smad3 are involved in the expressions and activations of molecules in IPF, which stimulate lung tissue to synthesize an amount of synthetic collagen, leading to an imbalance of extracellular matrix (ECM) regulation and resulting in ECM [58, 59]. Our experiment demonstrated that Schisandra inhibited pulmonary fibrosis as mediated by TGF-*β*1/Smad3/4 signaling pathways. Schisandra inhibited the expression of TGF-*β*1 and reduced the activation of T*β*RI and downregulated the expressions of Smad3 mRNA and Smad4 mRNA to depress the production of Smad3 and Smad4, thus preventing the binding between Smad3 and T*β*RI from producing dimer and the binding between the dimer with Smad4. Blocking the TGF-*β*1/Smad pathway inhibited AM polarization to the M2 phenotype. Smad7 negatively regulates the TGF-*β*1/Smad pathway, thereby interfering with the binding of Smad3 and Smad4 [60]. In vitro, Schisandra promoted the expression of Smad7, which also negatively regulated the TGF-*β*1/Smad pathway to inhibit the polarization of the M2. Based on these investigations, we conclude that Schisandra inhibits the polarization of the M2 in the rats with bleomycin-induced pulmonary fibrosis. And our attempt provides new evidence for the potential therapeutic effects of Schisandra on fibrotic diseases.

## 5. Conclusion

In summary, our data suggest that Schisandra inhibited the progress of IPF; it suppressed the polarization of M2 macrophages via the TGF-*β*1/Smad signaling pathway. Besides, this finding enriches our understanding of the effect of Schisandra on pulmonary fibrosis, which may be an experimental basis for future clinical application.

## Figures and Tables

**Table 1:** The results of alveolitis and fibrosis severity classification at different time points.

Groups	Alveolitis (Day 7)					Fibrosis (Day 28)				
	*N*	(0)	(+)	(++)	(+++)	*N*	(0)	(+)	(++)	(+++)
Sham	8	7	1	0	0	8	7	1	0	0
Model	8^#^	0	1	1	6	8^#^	0	0	2	6
Schisandra	8^#^^∗^	0	1	3	4	8^#^^∗^	0	3	3	2
Dex	8^#^^∗^	0	3	4	1	8^#^^∗^	0	2	4	2

Note: on the stage of alveolitis (Day 7), “(0)” indicates no obvious alveolar rupture and fusion; “(+)” indicates mild alveolar rupture and fusion; “(++)” indicates moderate alveolar rupture and fusion; “(+++)” indicates severe alveolar rupture and fusion; on the stage of alveolitis (Day 28), “(0)” indicates no obvious fibrous tissue hyperplasia; “(+)” indicates mild fibrous tissue hyperplasia; “(++)” indicates moderate fibrous tissue hyperplasia; “(+++)” indicates severe fibrous tissue hyperplasia. ^#^*P* < 0.05 vs. sham; ^∗^*P* < 0.05 vs. model.

## Data Availability

The data and materials supporting the conclusions of this article are included in the article.
